# THE REVIEW PROCESS AND TAKING STEPS TOWARD A SUCCESSFUL APPLICATION

**DOI:** 10.1093/geroni/igz038.764

**Published:** 2019-11-08

**Authors:** Elia Femia, Dana Plude

**Affiliations:** 1 National Institutes of Health, Bethesda, Massachusetts, United States; 2 National Institute on Aging, Bethesda, Maryland, United States

## Abstract

Applications reviewed by the Center for Scientific Review go through a process that starts with assignment to a review panel and ends with the assignment of a priority score and the production of a written evaluation (summary statement). This process is overseen by a Scientific Review Officer, who enlists expert reviewers to evaluate the scientific and technical merit, and the potential contribution and impact of the application to the research field. The goal is to ensure that applications are expertly and fairly evaluated in accordance to the NIH policies of rigor, reproducibility, transparency, and research integrity. In this presentation, learn about the criteria for review of an application, how reviewers shape their evaluation, and what applicants should be addressing in their grant applications. From the field, hear tips from successful applicants and experienced reviewers about what they do and what they look for in a well-written application.

